# Esophageal impedance planimetry during per-oral endoscopic myotomy guides myotomy extent

**DOI:** 10.1007/s00464-024-11067-4

**Published:** 2024-07-23

**Authors:** Ali M. Kara, A. J. Haas, Hemasat Alkhatib, Jamie DeCicco, Ramiro Cadena Semanate, Hee Kyung (Jenny) Kim, Rachna Prasad, Sergio Bardaro, Amelia Dorsey, Kevin El-Hayek

**Affiliations:** 1 Department of Surgery, The MetroHealth System, 2500 MetroHealth Drive, Cleveland, OH 44109 USA; 2 Case Western Reserve University School of Medicine, 10900 Euclid Ave, Cleveland, OH 44106 USA; 3 Northeast Ohio Medical University, 4209 St. Rt. 44, Rootstown, OH 44272 USA; 4 Cleveland Clinic Lerner College of Medicine, 9501 Euclid Ave, Cleveland, OH 44195 USA

**Keywords:** FLIP technology, Achalasia, Impedance planimetry, Distensibility index, Per oral endoscopic myotomy

## Abstract

**Introduction:**

Peroral endoscopic myotomy (POEM) is the standard treatment for achalasia. Functional luminal imaging probe (FLIP) technology enables objective measurement of lower esophageal sphincter (LES) geometry, with literature linking specific values to improved post-POEM outcomes. Our study assesses FLIP’s intraoperative use in evaluating myotomy extent in real-time.

**Methods:**

Retrospective data from all patients undergoing POEM with intraoperative FLIP measurements were extracted from June 2020 to January 2023. The primary endpoint was intraoperative FLIP measurements, management changes, and symptom improvement (Eckardt score).

**Results:**

Fourteen patients (age 56 ± 14 years, BMI 28 ± 7 kg/m^2^) were identified. Most patients were female (64%). Predominantly, patients presented with type II achalasia (50%). FLIP measurements were taken before and after myotomy, demonstrating increases in mean distensibility index (DI) 1.6 ± 1. 4 to 5.4 ± 2.1 mm^2^/mmHg (*p* < 0.05) and mean diameter (Dmin) 6 ± 1.8 to 10.9 ± 2.3 mm (*p* < 0.05) at 50 ml balloon fill. Additional myotomy was performed in one patient when an inadequate increase in FLIP values were noted. Mean operative time was 98 ± 28 min, and there were no intraoperative complications. At the 30-day follow-up, median Eckardt score decreased from mean a preoperative score of 7 ± 2 to a post-operative mean of 2 ± 3, with 10 patients (78%) having a score ≤ 2. In total, four patients experienced symptom recurrence, with repeat FLIP values revealing a significant decrease in DI from 7 ± 2.2 post-POEM to 2.5 ± 1.5 at recurrence. FLIP technology identified LES pathology in 3 out of 4 (75%) patients, facilitating referral to LES-directed therapy.

**Conclusion:**

Our study adds to the literature supporting the use of FLIP technology during the POEM procedure, with most patients achieving ideal values after a standard-length myotomy. This suggests the potential benefits of shorter myotomies guided by FLIP to achieve comparable outcomes and reduce postoperative GERD risk. Collaborative standardization of study designs and outcome measures is crucial for facilitating prospective trials and cross-setting outcome comparisons.

**Graphical abstract:**

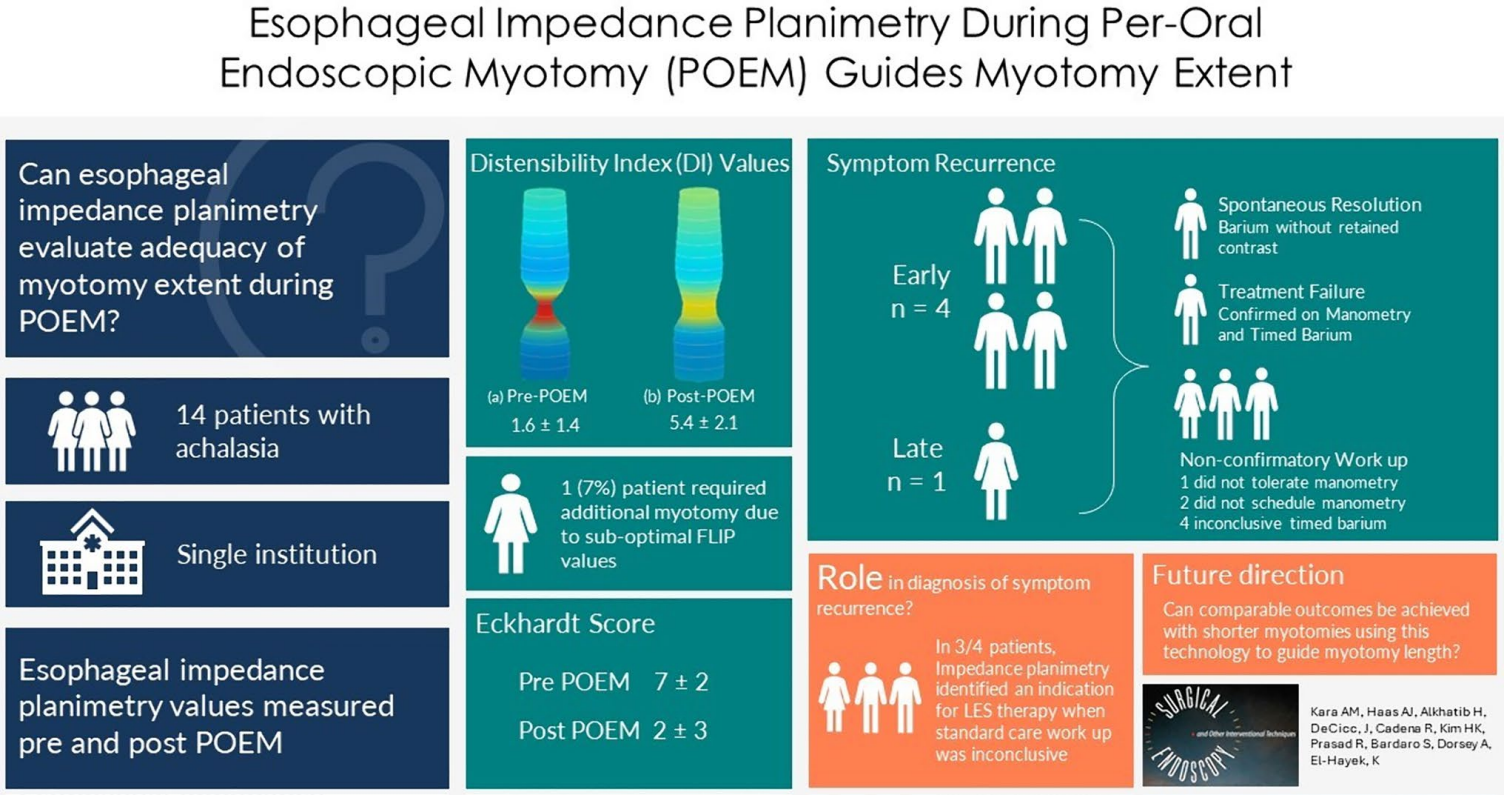

**Supplementary Information:**

The online version contains supplementary material available at 10.1007/s00464-024-11067-4.

Achalasia is a rare esophageal motility disorder characterized by insufficient relaxation of the lower esophageal sphincter and impaired or absent normal esophageal peristalsis. Treatment options for achalasia, whether endoscopic or surgical, aim to reduce the basal and swallow-induced sphincter pressures to provide relief by lowering the resistance to flow at the lower esophageal sphincter [1], achieved through performing a myotomy. Laparoscopic Heller myotomy with fundoplication has traditionally been the preferred treatment due to its longer-lasting effects compared to endoscopic balloon dilatation in patients who could tolerate surgery [2]. More recently, the peroral endoscopic myotomy (POEM) technique has emerged as an alternative to laparoscopic Heller myotomy with fundoplication, offering clinical success while avoiding major abdominal surgery [3]. However, due to high reflux rates reported post-operatively, there continues to be interest in augmenting the assessment of the myotomy to mitigate this complication.

The functional lumen imaging probe (FLIP) using the Endoflip™ catheter (Medtronic, Minneapolis, MN) employs impedance planimetry sensors to acquire precise geometric measurements of the lower esophageal sphincter, aiding in achalasia diagnosis, treatment endpoint definition, and intraoperative decision-making through real-time assessment of lower esophageal sphincter geometry [4, 5]. Previous studies have shown that FLIP-guided myotomy leads to improved outcomes, with specific parameter values correlating with treatment success [6, 7]. Additionally, some studies have identified values associated with post-procedural reflux [6, 8]. These findings suggest the potential benefit of targeting specific values using FLIP technology to optimize patient outcomes. Given these findings, our institution has integrated FLIP technology into our POEM procedure and follows prior literature in assessing treatment endpoints for patients. This study aims to present our experience with FLIP technology during POEM procedures, focusing on intraoperative decision-making guided by changes in FLIP values, postoperative outcomes, and associated alterations in FLIP values.

## Materials and methods

### Study design and patient selection

This was a retrospective study that was approved by the Institutional Review Board (IRB). All data was obtained through review of electronic medical records. Our patient population included all patients who underwent POEM for the diagnosis of achalasia with intraoperative use of FLIP technology between 2020 and 2023. All procedures were conducted by a single surgeon (KME) at a safety-net metropolitan anchor hospital with academic affiliations. The STROBE statement checklist was used to present this study [9].

### Study variables and outcomes

The primary objective of this study was to investigate the use of FLIP technology to assess the changes in the geometry of the lower esophageal sphincter (LES) during POEM. The main outcomes focused on assessing pre- and post-myotomy FLIP values and any intraoperative adjustments made based on FLIP measurements. FLIP values obtained included the distensibility index (DI, mm^2^/mmHg) as well as the minimum diameter (D_min_, mm), measured at 40, 50, and 60 ml balloon fill volume. DI values reflect the compliance of the LES, with high values indicating more compliance, and lower values indicating less compliance, indicative of achalasia-type pathology.

Secondary outcomes included postoperative treatment response as measured using changes in patient-reported Eckardt score. The Eckardt Symptom Score is based on four categories: dysphagia, retrosternal chest pain, regurgitation, and weight loss, with a maximum score of 12. A score of 3 or higher indicates poor symptom control, while a score below 3 indicates successful treatment [10]. At our institution, it is our standard protocol to administer this questionnaire preoperatively, postoperatively at 1-month follow-up, and again at 6–12 months follow-up. Other secondary outcomes included post-operative complications collected as binary (yes/no), including persistent reflux requiring medication, leak, and any major cardiopulmonary complications. These outcomes were measured at two time points: 30-day follow-up and 6–12 months follow-up. Additional secondary outcomes included assessment of recurrent symptoms, treatment failure and the utility of FLIP technology in the evaluation of these patients. Early recurrence of symptoms was defined as occurring within the 30-day follow-up window, while late recurrence was defined as occurring within the 6–12 months follow-up window.

Patient demographic data included age, body mass index (BMI), gender, ethnicity, insurance status, smoking status, Chicago Classification v3.0 and v4.0 for achalasia, comorbidities, history of prior intervention including dilation or Botox injection, and American Society of Anesthesiology performance classification (ASA). Intraoperative variables included mucosotomy site (anterior or posterior) and operative time in minutes.

### Preoperative work up and POEM technique

All patients underwent preoperative high-resolution manometry and timed barium swallow to diagnose achalasia and identify its type. The POEM procedure was performed with the patient positioned supine under general endotracheal anesthesia. To measure FLIP values, we followed previously published experience-based expert consensus guidelines for using FLIP technology during POEM procedures [5]. A 16 cm catheter was used, and pre-myotomy values were obtained at balloon fills of 40 ml, 50 ml, and 60 ml. The catheter was then removed, and the submucosa was injected with a mixture of saline and a submucosal lifting agent. The esophageal length was measured, and a point 10–12 cm above the esophagogastric junction was chosen to create the mucosotomy. An endoscopic tunnel was created in the submucosal plane and extended to the proximal stomach. A myotomy of the circular muscle fibers was then made using the triangle tip electrosurgical knife (KD-640L) in a proximal to distal fashion, using the endoscope to measure the length of the myotomy. The myotomy was preferentially done posteriorly unless challenges such as food pooling or submucosal fibrosis necessitated an anterior myotomy. Next, an 8–10 cm proximal myotomy along the esophagus was performed with an extension of 3–4 cm distal to the esophagogastric junction (EGJ) along the proximal stomach. The FLIP catheter was then reintroduced, and post-myotomy values were measured at balloon fills of 40 ml, 50 ml, and 60 ml. If DI values indicated adequate myotomy (DI 4–8 mm^2^/mmHg), the catheter was removed. If DI values indicate inadequate myotomy, then the myotomy was extended distally or proximally, and values were reobtained. The mucosotomy was then sealed with endoscopic clips, and the procedure was completed.

### Study analysis

Descriptive statistics were utilized to analyze the data, including frequencies, percentages, medians, interquartile ranges, means, and standard deviation, where appropriate to summarize the results. Wilcoxon matched-pairs signed-rank test was used to assess changes in FLIP measurements and Eckardt score before and after myotomy. Missing data points and patients lost to follow-up were reported.

## Results

### Patient demographics and preoperative details

Fourteen patients underwent POEM with intraoperative FLIP guidance during the study period from June 2020 to January 2023. The patient demographics and preoperative details are presented in Table 1. The majority of the patients in this study were females (9, 64%), had Medicare insurance (7, 50%), and were classified as American Society of Anesthesiologists class III (9, 64%). The most common achalasia type was type II (7, 50%). The mean preoperative Eckardt score was 7 ± 2.

**Table 1:** Patient demographics and preoperative details

Total patients, *N*	14	
Age, years, (mean ± SD)	56	±14
BMI, (mean ± SD)	28	±7
Female, *N* (%)	9	64%
Race		
Caucasian	7	50%
African American	4	29%
Hispanic	2	14%
Asian	1	7%
Insurance		
Private	2	14%
Medicare	7	50%
Medicaid	4	29%
Self-pay	1	7%
Comorbidities		
GERD	11	79%
Type II diabetes mellitus	4	29%
OSA	5	36%
Peptic ulcer disease	1	7%
Hypertension	9	64%
Coronary artery disease	1	7%
Psychiatric history	5	36%
Autoimmune	0	0%
Current or Former smoker	6	46%
Prior intervention		
Prior POEM	0	0%
Botox	2	14%
Endoscopic balloon dilation	2	14%
Pneumatic dilation	1	7%
Heller myotomy	0	0%
ASA class		
2	3	21%
3	9	64%
4	2	14%
Preop Eckardt score (mean ± SD)	7	±2
Achalasia stage		
1	4	29%
2	7	50%
3	1	7%
EGJOO	2	14%

*BMI* body mass index, *EGJOO* esophagogastric junction outflow obstruction, *GERD* Gastroesophageal reflux disease, *OSA* obstructive sleep apnea, *ASA* American Society of Anesthesiology

### Operative details and intraoperative changes in FLIP values

**Fig. 1:**
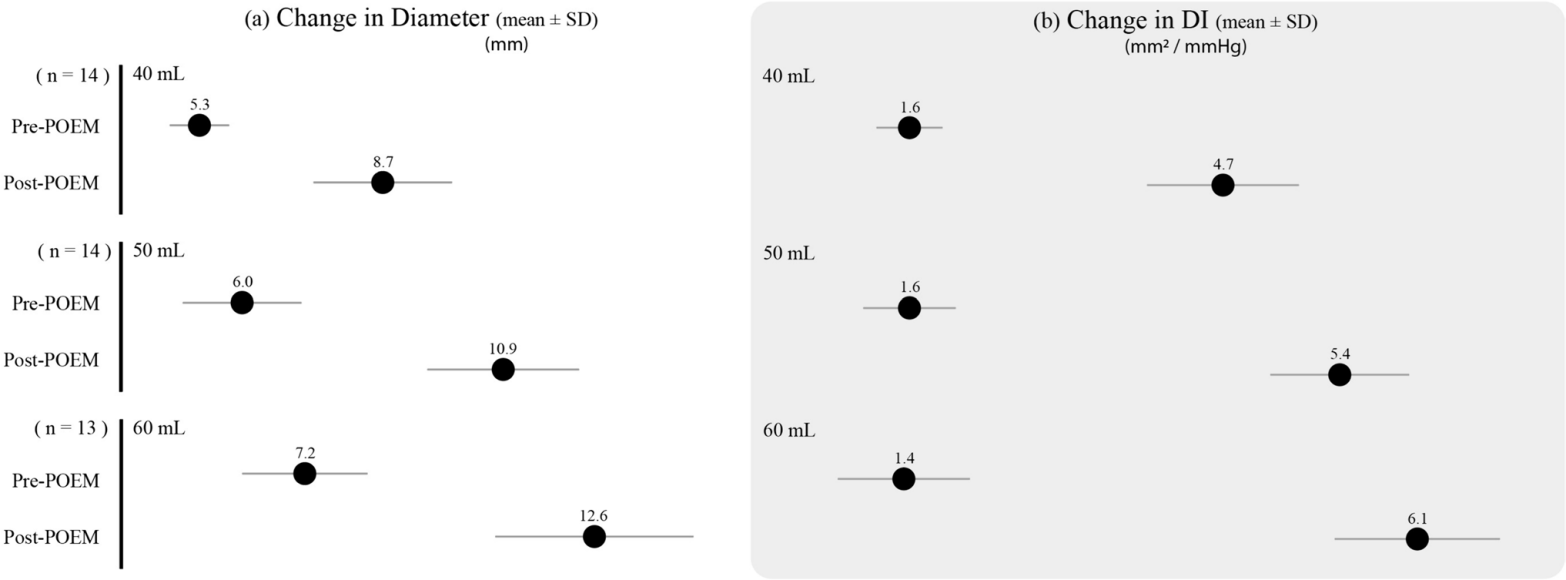
FLIP values obtained pre-POEM and post-POEM: **a** change in minimum diameter (D_min_, mm); and **b** change in distensibility index (DI, mm^2^/mmHg)

The location of myotomy was posterior in 86% of cases, and anterior in 14% of cases. The mean operative time was 98 ± 27 min, and no intraoperative complications were noted. FLIP measurements were obtained before and after myotomy, demonstrating significant improvement as follows: mean DI 1.6 ± 1. 4 vs. 5.4 ± 2.1 mm^2^/mmHg (*p* < 0.05), mean D_min_ 6 ± 1.8 vs. 10.9 ± 2.3 mm (*p* < 0.05) at 50 ml balloon fill. Figure 1 demonstrates changes in FLIP values pre-myotomy and post-myotomy for all balloon fill volumes. Only 1/14 (7%) patients required additional myotomy due to suboptimal values. After initial myotomy, the DI values for this patient increased from 1.4 to 3.4 mm^2^/mmHg, while D_min_ increased from 8.5 to 11 mm (60 ml balloon volume). To aim for DI values closer to 4, the surgeon opted for an additional 1–2 cm myotomy. At the completion of the additional myotomy, DI values did not significantly change with a final value of 2.9 mm^2^/mmHg, while D_min_ increased to 12 mm, elevating the risk of reflux. Consequently, no further myotomy was performed.

### Post-operative outcomes

All patients were discharged on a full liquid diet for two weeks and twice daily proton pump inhibitors, to be taken for at least three months. All patients followed up at a mean of 20 ± 5 days for their 30-day follow-up. Eckardt score was not completed by one patient at this time point. Total Eckardt score decreased from a preoperative mean of 7 ± 2 to 2 ± 3 (*p* < 0.05), with ten patients (78%) having an Eckardt score of ≤ 2, and three patients (23%) reporting Eckardt scores > 3.

Of the fourteen patients, two (14%) experienced postoperative complications. One, with a history of DVT/PE, developed a PE, while the other had a submucosal tunnel leak, which was resolved without surgery through bowel rest and placement of a distal nasoenteric feeding tube. Additionally, 4/14 (29%) of patients reported symptoms of dysphagia and oral intolerance, including the patient who required an additional myotomy based on FLIP values.

A total of 11/14 (79%) had long-term follow-up at a mean of 15 ± 9 months. At that time point, 1 (7%) patient reported recurrent symptoms of dysphagia and oral intolerance.

### Symptom recurrence and treatment failure

**Fig. 2:**
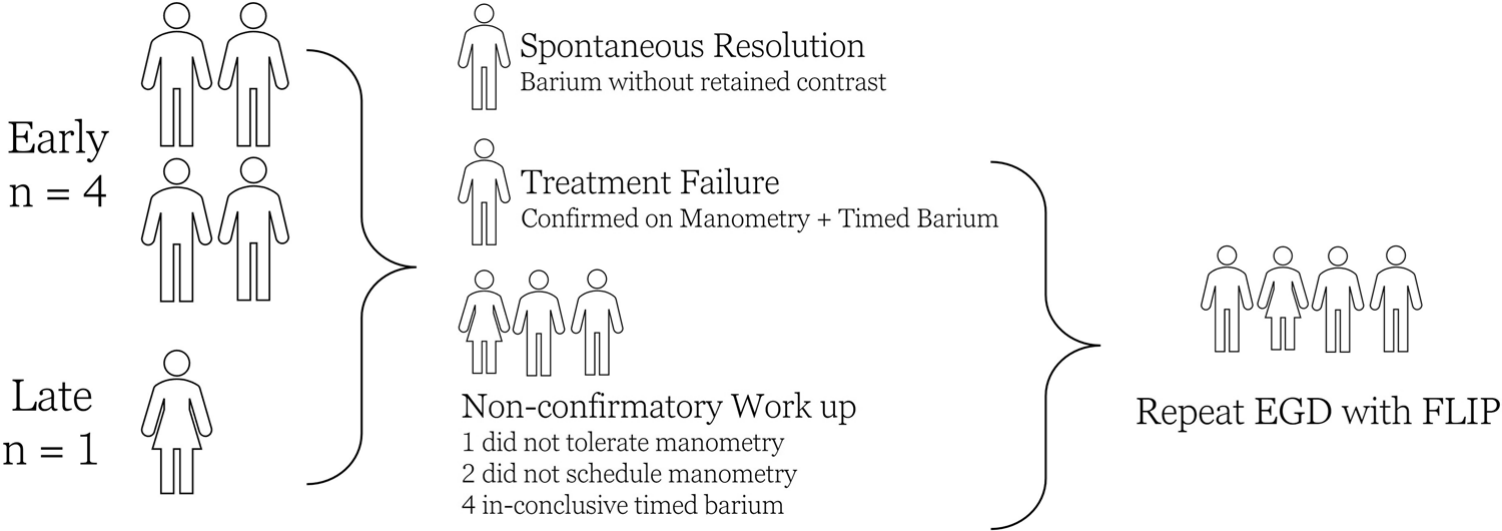
Graphical representation of patient symptom recurrence rates and diagnostic test outcomes

Four patients (29%) reported symptom recurrence early postoperatively, while one (7%) reported late recurrence. All patients were advised to undergo timed barium swallow and manometry. One patient with early recurrence had normal results on timed barium swallow and experienced symptom resolution by 2 months follow-up without any further intervention. Another patient, diagnosed with treatment failure based on timed barium and manometry findings indicative of achalasia, was referred for endoscopy with FLIP. The remaining three patients had inconclusive workups: one could not tolerate manometry, while three did not schedule the test. All underwent a timed barium swallow with findings that are inconclusive. They were subsequently referred for repeat endoscopy with FLIP. Figure 2 presents a graphical representation of patient symptom recurrence rates and diagnostic test outcomes.

Subsequent FLIP assessments for the four patients showed a significant decrease in DI, dropping from a mean of 7 ± 2.3 post-POEM to 2.5 ± 1.5 at the recurrence time point, without significant change in diameter with a mean of 11.5 ± 2.2 post-POEM to 11.3 ± 1.9 at the recurrence time point (50 ml balloon volume). The patients were considered suitable candidates for LES-directed therapy, which involved serial botox injections and dilation. Three patients experienced symptom improvement on follow-up but reported severe reflux, necessitating the initiation of medical therapy. This included the patient who required additional myotomy based on FLIP values during their POEM procedure. One patient remained refractory to treatment and subsequently required percutaneous feeding tube placement for nutritional support.

**Fig. 3:**
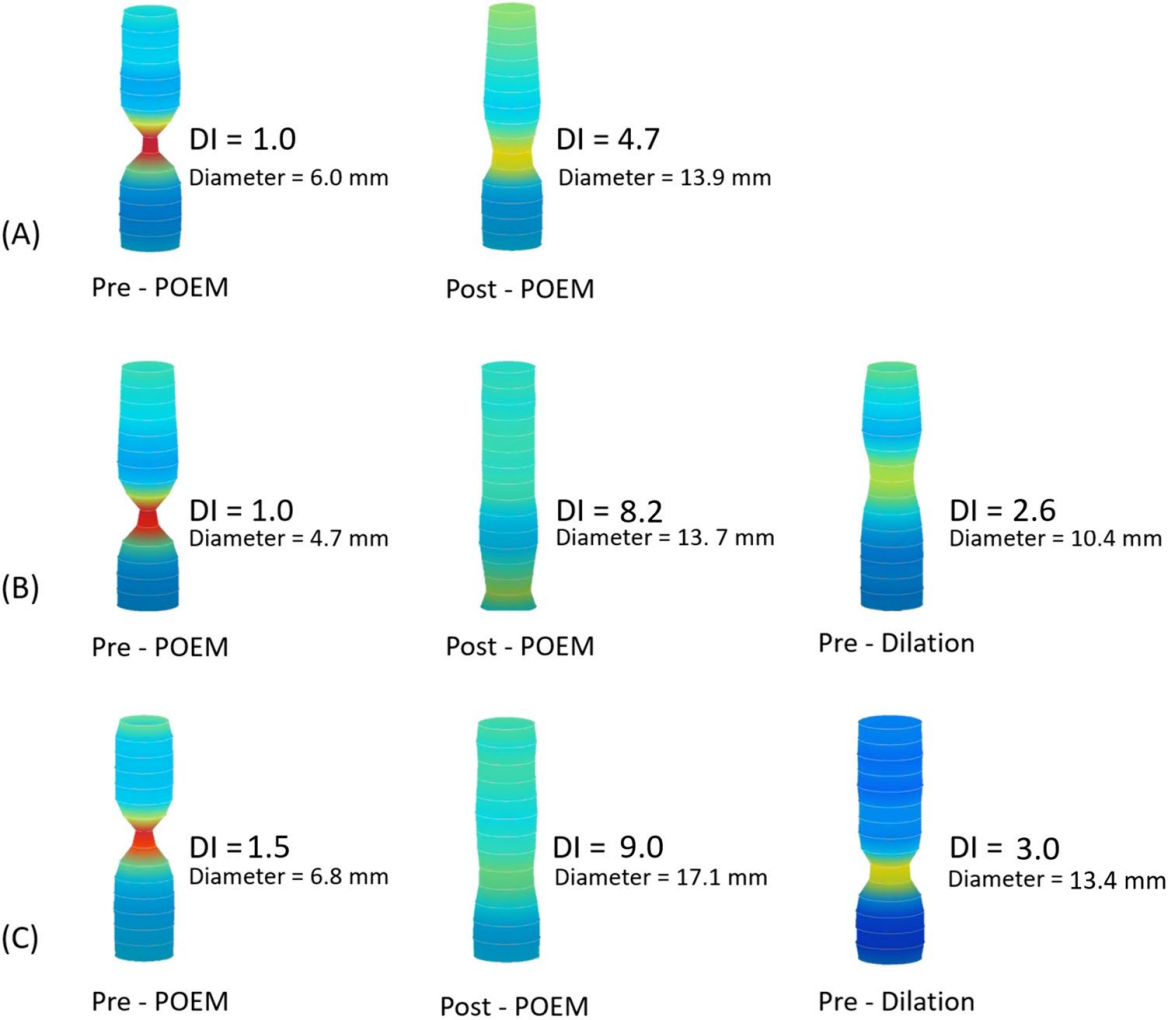
Illustration demonstrating dynamic changes to FLIP values intraoperatively in three patient examples undergoing POEM procedure (**A**–**C**), and the FLIP values in those with recurrent symptoms (**B**, **C**)

Figure 3 illustrates intraoperative FLIP values in three patient examples undergoing POEM procedure (A, B, and C), along with changes in values for two patients experiencing recurrent symptoms (B and C).

## Discussion

Our study evaluates the utility of FLIP technology during POEM procedures to assess myotomy adequacy, focusing on values previously shown in the literature to be associated with favorable clinical outcomes and reduced postoperative GERD risk. Post-POEM FLIP values met ideal criteria in the majority of patients undergoing standard of care POEM at our institute. Additional myotomy was required in only 1 out of 14 cases (7%) to achieve optimal values. Most patients exhibited symptom improvement, as evidenced by a reduction in the Eckardt score. Among those experiencing recurrent symptoms, FLIP values did not consistently predict this outcome. Finally, FLIP technology proved beneficial in identifying potential candidates for LES-directed therapy when conventional diagnostic tests (Manometry + Timed Barium) were unavailable or inconclusive, facilitating patient counseling and guiding interventions such as dilation.

The application of FLIP technology in myotomy procedures (surgical or endoscopic) has garnered significant interest due to its ability to provide objective values for assessing changes in esophagogastric junction (EGJ) geometry and identifying target values associated with improved outcomes. Holmstrom et al. compared populations undergoing POEM with and without FLIP technology, revealing a need for additional myotomy in over half of FLIP-guided cases to target DI values, which was overall associated with higher clinical success rates (93% vs. 81%) [7]. Our study observed a significantly lower rate, with only 7% necessitating additional myotomy. Discrepancies in the standard of care myotomy and the utilization of FLIP technology may have influenced this outcome.

Previous studies have noted similar improvement in outcomes correlated with specific FLIP values [6, 11, 12], although there is significant variability in the values studied and the subsequent cut-off that was associated with these outcomes. Overall, based on the previously mentioned studies, ideal DI values typically range between 4 and 8 mm^2^/mmHg, with values < 3 correlating to treatment failure. In our cohort, patients experiencing symptom recurrence post-myotomy had an initial Post-POEM mean DI of 7 ± 2.2 at 60 ml balloon volume, with none demonstrating a post-myotomy DI < 3. Consequently, the predictive capability of intraoperative FLIP values for treatment failure remains ambiguous in our population, aligning with studies that found no outcome differences associated with intraoperative FLIP values [13, 14]. Amundson et al. recently utilized FLIP technology to explore EGJ compliance rather than DI and noted its superior performance in identifying ideal values linked to improved postoperative outcomes [15]. Despite these insights, the consistency of FLIP technology’s utility in POEM remains variable and necessitates further investigation, likely due to the complexity of the pathology under study and the diverse geometric parameters available through FLIP technology that can serve as proxies to correlate with improved outcomes.

Another potential advantage of FLIP technology is the ability to identify values that are associated with postoperative reflux. Attaar et al. linked final DI values > 2.7 and cross-sectional area > 83 to postoperative esophagitis on endoscopy, although this did not correlate with differences in patient-reported symptom severity or quality of life measures [16]. Teitelbaum et al., in their study, categorized patients into thirds and observed that those with the highest DI (> 9) were more prone to report a GERD-HRQL score > 7 [11]. Su et al. found no direct association between final DI values but noted that patients with a final cross-sectional area > 96 or D_min_ of 11 were more likely to have high RSI scores during follow-up [6]. In our cohort, no patients required long-term medication for GERD following the POEM procedure. However, all four patients who underwent dilation and botox for recurrent symptoms developed GERD and subsequently received medical therapy. Nevertheless, interpreting these findings is challenging as the data collection for this variable was limited to a binary yes/no during our follow-up visits without incorporating quality-of-life questionnaires or symptom severity scores.

Finally, our study explores the role of FLIP technology in evaluating symptom recurrence following POEM. In cases where manometry was not feasible, and timed barium swallow results were inconclusive, identifying DI scores indicative of low compliance LES prompted LES-directed therapy. This was applicable to 3 out of 4 (75%) patients reporting symptom recurrence. This was similarly found by a multi-institutional study evaluating the use of FLIP technology in treatment failure patients [17], in which 60% of patients had normal findings on manometry, of which 7% were found to have abnormal DI values using FLIP, prompting referral for LES-directed therapy. Importantly, the role of FLIP technology in assessing the efficacy of LES-directed therapy still needs to be explored, resulting in uncertainty about its current application in those settings.

It is crucial to acknowledge several limitations inherent in this study that warrant consideration when interpreting the findings. Firstly, the sample size is small, and the study was conducted at a single center, limiting generalizability and restricting the ability to make robust outcome comparisons or establish optimal ranges for improved outcomes. Moreover, being retrospective in nature, the study is subject to inherent biases and limitations influenced by confounding factors that were not adjusted for given the small sample size. Furthermore, our assessment of reflux relied on a binary yes/no determination based on the need for medication, serving as a proxy for identifying significant reflux impacting quality-of-life. We did not employ quality of life questionnaires such as RSI and GERD-HRQL, limiting comparisons with existing literature.

In summary, the integration of FLIP technology has undoubtedly added complexity to the evaluation, treatment, and postoperative assessment of achalasia. Yet, inconsistencies persist in the literature due to the disease’s intricate nature and the variability in FLIP-derived geometric values used as outcome proxies. Our study adds to the expanding evidence supporting the use of this technology in this patient population. We advocate for the integration of FLIP technology by other foregut surgeons to advance our understanding across diverse patient populations and institutions. We found that a POEM procedure with standard length myotomy resulted in FLIP values within ideal ranges for most patients. This prompts the question of whether comparable outcomes can be achieved with shorter myotomies, as there is currently no literature addressing this issue. The objective would be to target FLIP values that mitigate reflux while ensuring the resolution of achalasia symptoms. We suggest leveraging expert consensus across multiple institutions to develop specific study designs and standardized outcome measures, facilitating prospective randomized controlled trials and enabling outcome comparisons across diverse settings.

### Supplementary Information

Below is the link to the electronic supplementary material.

Supplementary file1 (DOCX 15 KB)
