# Supplementary material for: Esophageal impedance planimetry during per-oral endoscopic myotomy guides myotomy extent
Source: Surg Endosc. 2024 Jul 23;38(9):5377–84. doi: 10.1007/s00464-024-11067-4 (PMC11362179; doi:10.1007/s00464-024-11067-4)
Supplement: Supplementary file 1 — Supplementary file1 (DOCX 15 KB) [file 464_2024_11067_MOESM1_ESM.docx]

Supplemental Table 1: Distensibility index and minimal diameter values pre and post POEM

| Balloon fill volume | FLIP values | Pre POEM | Post POEM |
| --- | --- | --- | --- |
| 40ml (n=14) | DI | 1.6 ± 1.0 | 4.7 ± 2.3 |
|  | D_min_ | 5.3 ± 0.9 | 8.7 ± 2.1 |
| 50ml (n=14) | DI | 1.6 ± 1.4 | 5.4 ± 2.1 |
|  | D_min_ | 6.0 ± 1.8 | 10.9 ± 2.3 |
| 60ml (n=13) | DI | 1.4 ± 1.0 | 6.1 ± 2.5 |
|  | D_min_ | 7.2 ± 1.9 | 12.6 ± 3.0 |
